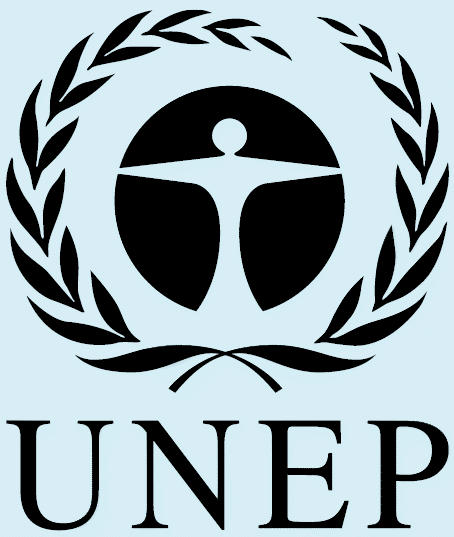# EHPnet: UNEP.Net Freshwater Portal

**Published:** 2005-07

**Authors:** Erin E. Dooley

Even though 70% of the Earth’s surface is covered with water, little of that is freshwater. Today, one-third of the world’s population lives in countries with moderate or high water stress, a fact that leads many experts to proclaim that water may possibly be the primary cause of international tensions and the foremost threat to environmental health in the twenty-first century. The United Nations Environment Programme (UNEP) has set up a Freshwater Portal, located online at http://freshwater.unep.net/, as a centralized resource for anyone looking to learn more about freshwater use, resources, and scarcity. The fully searchable site is part of UNEP’s United Nations Environment Network, which aims to bring specialized environmental science communities together under one umbrella.

The issues associated with freshwater resources are wide-ranging. Many surface water sources are shrinking, as population growth fuels desertification and overuse of resources. Several large rivers now run dry for at least part of the year, and lakes are shrinking. Groundwater, too, is being affected by pollution, salinization, and overuse. Overpumping of water is causing large areas to sink, including almost 60,000 square miles in China. Infrastructure also is in crisis in many areas. In 2002, only 52% of people worldwide were connected to water systems, and only 30% were connected to sanitation services. Each year more than 5 million people die from water-related diseases, and diarrheal diseases are the leading cause of death in children.

The Freshwater Portal has been indexed by nine key issues. These include water scarcity, irrigated agriculture, water and sanitation, water quality, groundwater, transboundary water management, water and ecosystems, floods and droughts, and urban water. For each key issue, UNEP has collected relevant reports, background papers, websites, and other resources. For example, the Water Scarcity section includes links to papers on balancing water uses, managing water within agriculture, and the relationship between water scarcity and poverty. The Water and Sanitation section provides links to two reports as well as to the World Bank Water and Sanitation Program, which guides international efforts to build infrastructure in this area. And the Groundwater section links to a global overview of ground-water conditions, which goes on to detail best management practices for this resource.

The site is also cross-indexed by resource type. Visitors can view all case studies/best practices documents, for example, or go directly to conference proceedings covering multiple topic areas.

Similar portals on other topics are accessible from the top of the homepage. Visitors can choose other Thematic Portals, such as Climate Change or Urban Environment, and can also select from Regional Portals to view information specific to the Arctic, Europe, or Latin America. The homepage also highlights global water assessments and announcements of recent documents, statements, and meetings. Also included is a link to Earthprint.com, where visitors can purchase freshwater publications produced by UNEP and other international organizations.

## Figures and Tables

**Figure:**